# 4-Sulfamoylanilinium nitrate

**DOI:** 10.1107/S1600536811038827

**Published:** 2011-09-30

**Authors:** S. Pandiarajan, S. Balasubramanian, B. Ravikumar, S. Athimoolam

**Affiliations:** a Department of Physics, Devanga Arts College, Aruppukottai 626 101, India; b Department of Physics, University College of Engineering Nagercoil, Anna University of Technology Tirunelveli, Nagercoil 629 004, India

## Abstract

In the crystal structure of the title compound, C_6_H_9_N_2_O_2_S^+^·NO_3_^−^, the cations and anions are connected by N—H⋯O hydrogen bonds into a three-dimensional network.

## Related literature

For the biological importance of the title compound, see: Kent (2000 ▶). For related structures, see: Alléaume & Decap (1965*a* ▶,*b* ▶); Buttle *et al.* (1936 ▶); Chatterjee *et al.* (1981 ▶); Gelbrich *et al.* (2007 ▶, 2008 ▶); Gelmboldt *et al.* (2004 ▶); Hughes *et al.* (1999 ▶); O’Connell & Maslen (1967 ▶); O’Connor & Maslen (1965 ▶); Smith *et al.* (2001 ▶); Zaouali Zgolli *et al.* (2010 ▶). For the polymorphism of sulfanilamide, see: Burger (1973 ▶). For hydrogen-bond motifs, see: Etter *et al.* (1990 ▶).
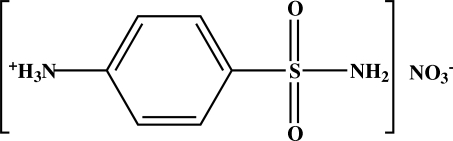


## Experimental

### 

#### Crystal data

C_6_H_9_N_2_O_2_S^+^·NO_3_^−^
*M* *_r_* = 235.22
Monoclinic, 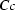

*a* = 14.1489 (19) Å
*b* = 8.1786 (11) Å
*c* = 8.6931 (12) Å
β = 107.129 (2)°
*V* = 961.3 (2) Å^3^
*Z* = 4
Mo *K*α radiation
μ = 0.34 mm^−1^
*T* = 293 K
0.24 × 0.22 × 0.19 mm

#### Data collection

Bruker SMART APEX CCD area-detector diffractometer
4345 measured reflections
1694 independent reflections
1689 reflections with *I* > 2σ(*I*)
*R*_int_ = 0.017

#### Refinement

*R*[*F* ^2^ > 2σ(*F* ^2^)] = 0.025
*wR*(*F* ^2^) = 0.061
*S* = 1.15
1694 reflections
157 parameters
2 restraints
H atoms treated by a mixture of independent and constrained refinement
Δρ_max_ = 0.17 e Å^−3^
Δρ_min_ = −0.25 e Å^−3^
Absolute structure: Flack (1983 ▶), 840 Friedel pairs
Flack parameter: 0.06 (5)

### 

Data collection: *SMART* (Bruker, 2001 ▶); cell refinement: *SAINT* (Bruker, 2001 ▶); data reduction: *SAINT*; program(s) used to solve structure: *SHELXTL/PC* (Sheldrick, 2008 ▶); program(s) used to refine structure: *SHELXTL/PC*; molecular graphics: *PLATON* (Spek, 2009 ▶); software used to prepare material for publication: *SHELXTL/PC*.

## Supplementary Material

Crystal structure: contains datablock(s) global, I. DOI: 10.1107/S1600536811038827/bt5645sup1.cif

Structure factors: contains datablock(s) I. DOI: 10.1107/S1600536811038827/bt5645Isup2.hkl

Supplementary material file. DOI: 10.1107/S1600536811038827/bt5645Isup3.cml

Additional supplementary materials:  crystallographic information; 3D view; checkCIF report

## Figures and Tables

**Table 1:** Hydrogen-bond geometry (Å, °)

*D*—H⋯*A*	*D*—H	H⋯*A*	*D*⋯*A*	*D*—H⋯*A*
N1—H1*A*⋯O2^i^	0.81 (3)	2.33 (3)	2.992 (2)	139 (2)
N1—H1*B*⋯O4^ii^	0.75 (3)	2.30 (3)	3.045 (3)	172 (3)
N2—H1*N*⋯O5^iii^	0.93 (3)	2.01 (3)	2.866 (2)	151 (3)
N2—H2*N*⋯O1^iii^	0.92 (2)	1.97 (2)	2.858 (2)	163 (2)
N2—H3*N*⋯O5^iv^	0.83 (3)	1.95 (3)	2.770 (2)	171 (2)

Symmetry codes: (i) 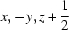; (ii) 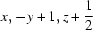; (iii) 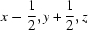; (iv) 
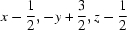
.

**Fig. 1.**
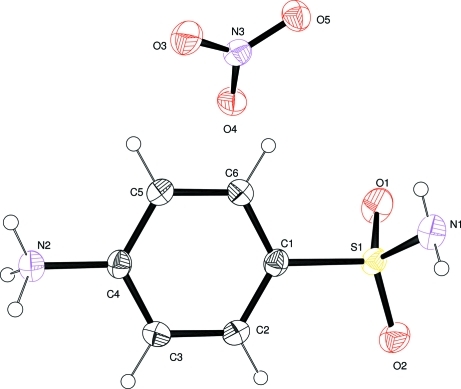
The molecular structure of the title compound with atom numbering scheme and 50% probability displacement ellipsoids.

**Fig. 2.**
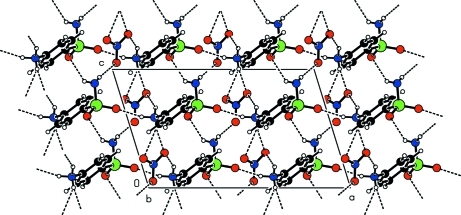
Packing diagram of the title compound viewed down the b-axis. H-bonds are shown as dashed lines.

## supplementary crystallographic information

### Comment

Sulfanilamide, a sulfonamide antibacterial, acts as competitive inhibitor of the enzyme dihydropteroate synthetase (DHPS), an enzyme involved in folate synthesis which involves *para*-aminobenzoic acid (PABA). PABA is needed in enzymatic reactions that produce folic acid which acts as a coenzyme in the synthesis of purine, pyrimidine and other amino acids (Kent, 2000). Sulfonamide drugs were the first antimicrobial drugs, and paved the way for the antibiotic revolution in medicine. The antibacterial activity of sulfanilamide, was first investigated by Buttle (Buttle *et al.*, 1936). The use of sulfanilamide was eclipsed by its prodrugs, the more effective sulfadrugs, shortly afterwards. From literature, it is observed that sulfadrugs are remarkably polymorphic. The polymorphs of sulfathiazole (Hughes *et al.*, 1999) and sulfapyridine (Gelbrich *et al.*, 2007) were already reported. The polymorphism of sulfanilamide was extensively investigated over a number of years (Burger, 1973). There are three well known polymorphs, usually represented as α, β and γ sulfanilamides (Alléaume & Decap, 1965*a*,*b*; O'Connor & Maslen, 1965; O'Connell & Maslen, 1967). Based on the above specifics, we are interested on the investigation of hydrogen bonding tendancy and its reactivity with different inorganic/organic acids.

The asymmetric part of the unit cell, contains a protonated sulfomylanilinium cation and a nitrate anion (Fig 1). The protonation on the one of the N sites is confirmed from C—N bond distance. The other geometrical parameters of the cation are in agreement with the reported structures of 4-homosulfanilamide hydrochloride (Chatterjee *et al.*, 1981), 4-aminobenzenesulfonamide (Gelbrich *et al.*, 2008), bis(4-Aminosulfonyl)benzeneammonium hexafluorosilicate (Gelmboldt *et al.*,2004), 4-sulfonamidoanilinium 3,5-dinitrosalicylate (Smith *et al.*, 2001) and 4-sulfamoylanilinium chloride (Zaouali Zgolli *et al.*, 2010).

The crystal structure is stabilized through intricate three dimensional hydrogen bonding network formed through N—H···O hydrogen bonds (Fig 2, Table 1). The N atom of the –NH_2_ group of the cation is hydrogen bonded with O atom of the S=O group making a zigzag chain C(4) motif extending along *c* axis of the unit cell (Etter *et al.*, 1990). Further, the N atom of the –NH_3_ group of the cation is hydrogen bonded with another O atom of the S=O group making a head-to-tail like chain C(8) motif extending along diagonal of the *ab*-plane. Nitrate anions are sandwiched between these two chains leading to a unusual asymmetric ring *R*_5_^5^(16) motif which involves four cation and one anion. Also, cations are linked through anion by two N—H···O hydrogen bonds [*viz*., N1—H1B···O4 (*x*, 1 - *y*, 1/2 + *z*) and N2—H3N···O5 (-1/2 + *x*, 3/2 - *y*, -1/2 + *z*)] forming a chain C_2_^2^(12)motif extending along diagonal of the *bc*-plane.

### Experimental

Colourless crystals of 4-sulfamoylanilinium nitrate suitable for single-crystal X-ray analysis were obtained by slow evaporation at room temperature from an aqeuous solution of sulphanilamide and nitric acid.

### Refinement

The H atoms bonded to N located were refined istropically.  All other H atoms were positioned geometrically and refined by the riding model approximation with d(C—H) = 0.93 Å and *U*_iso_(H)= 1.2 *U*_eq_(C).

### Crystal data

**Table:**

C_6_H_9_N_2_O_2_S^+^·NO_3_^−^	*F*(000) = 488
*M**_r_* = 235.22	*D*_x_ = 1.625 Mg m^−^^3^
Monoclinic, *C**c*	Mo *K*α radiation, λ = 0.71073 Å
Hall symbol: C -2yc	Cell parameters from 2432 reflections
*a* = 14.1489 (19) Å	θ = 2.3–24.3°
*b* = 8.1786 (11) Å	µ = 0.34 mm^−^^1^
*c* = 8.6931 (12) Å	*T* = 293 K
β = 107.129 (2)°	Block, colourless
*V* = 961.3 (2) Å^3^	0.24 × 0.22 × 0.19 mm
*Z* = 4	

### Data collection

**Table:**

Bruker SMART APEX CCD area-detector diffractometer	1689 reflections with *I* > 2σ(*I*)
Radiation source: fine-focus sealed tube	*R*_int_ = 0.017
graphite	θ_max_ = 25.0°, θ_min_ = 2.9°
ω scans	*h* = −16→16
4345 measured reflections	*k* = −9→9
1694 independent reflections	*l* = −10→10

### Refinement

**Table:**

Refinement on *F*^2^	Hydrogen site location: inferred from neighbouring sites
Least-squares matrix: full	H atoms treated by a mixture of independent and constrained refinement
*R*[*F*^2^ > 2σ(*F*^2^)] = 0.025	*w* = 1/[σ^2^(*F*_o_^2^) + (0.043*P*)^2^ + 0.0928*P*] where *P* = (*F*_o_^2^ + 2*F*_c_^2^)/3
*wR*(*F*^2^) = 0.061	(Δ/σ)_max_ = 0.001
*S* = 1.15	Δρ_max_ = 0.17 e Å^−^^3^
1694 reflections	Δρ_min_ = −0.25 e Å^−^^3^
157 parameters	Extinction correction: *SHELXTL/PC*, Fc^*^=kFc[1+0.001xFc^2^λ^3^/sin(2θ)]^-1/4^
2 restraints	Extinction coefficient: 0.050 (3)
Primary atom site location: structure-invariant direct methods	Absolute structure: Flack (1983), 840 Friedel pairs
Secondary atom site location: difference Fourier map	Flack parameter: 0.06 (5)

### Special details

**Table:**

Geometry. All e.s.d.'s (except the e.s.d. in the dihedral angle between two l.s. planes) are estimated using the full covariance matrix. The cell e.s.d.'s are taken into account individually in the estimation of e.s.d.'s in distances, angles and torsion angles; correlations between e.s.d.'s in cell parameters are only used when they are defined by crystal symmetry. An approximate (isotropic) treatment of cell e.s.d.'s is used for estimating e.s.d.'s involving l.s. planes.
Refinement. Refinement of *F*^2^ against ALL reflections. The weighted *R*-factor *wR* and goodness of fit *S* are based on *F*^2^, conventional *R*-factors *R* are based on *F*, with *F* set to zero for negative *F*^2^. The threshold expression of *F*^2^ > σ(*F*^2^) is used only for calculating *R*-factors(gt) *etc*. and is not relevant to the choice of reflections for refinement. *R*-factors based on *F*^2^ are statistically about twice as large as those based on *F*, and *R*- factors based on ALL data will be even larger.

### Fractional atomic coordinates and isotropic or equivalent isotropic displacement parameters (Å^2^)

**Table:**

	*x*	*y*	*z*	*U*_iso_*/*U*_eq_	
C1	0.29701 (12)	0.3638 (2)	0.66596 (19)	0.0342 (3)	
C2	0.20184 (13)	0.3652 (2)	0.5630 (2)	0.0403 (3)	
H2	0.1737	0.2705	0.5098	0.048*	
C3	0.14895 (12)	0.5103 (2)	0.5403 (2)	0.0428 (4)	
H3	0.0845	0.5138	0.4721	0.051*	
C4	0.19244 (12)	0.6488 (2)	0.6193 (2)	0.0334 (3)	
C5	0.28819 (14)	0.6483 (2)	0.7208 (2)	0.0414 (4)	
H5	0.3166	0.7437	0.7723	0.050*	
C6	0.34101 (13)	0.5037 (2)	0.7443 (2)	0.0424 (4)	
H6	0.4055	0.5005	0.8122	0.051*	
N1	0.39052 (16)	0.1289 (2)	0.8816 (2)	0.0511 (4)	
N2	0.13535 (11)	0.79981 (18)	0.59414 (18)	0.0374 (3)	
N3	0.55860 (12)	0.64779 (19)	0.6987 (2)	0.0447 (3)	
O1	0.45798 (12)	0.21351 (19)	0.6684 (2)	0.0596 (4)	
O2	0.30328 (10)	0.05499 (16)	0.60456 (18)	0.0520 (3)	
O3	0.51772 (13)	0.76152 (19)	0.7468 (2)	0.0658 (4)	
O4	0.53047 (11)	0.60273 (18)	0.55635 (17)	0.0557 (3)	
O5	0.63098 (11)	0.57493 (19)	0.79609 (15)	0.0522 (3)	
S1	0.36568 (3)	0.17908 (4)	0.69713 (4)	0.03650 (14)	
H1A	0.343 (2)	0.088 (3)	0.900 (3)	0.060 (7)*	
H1B	0.4231 (19)	0.194 (4)	0.932 (3)	0.056 (7)*	
H1N	0.156 (2)	0.875 (4)	0.678 (4)	0.074 (8)*	
H2N	0.0721 (18)	0.783 (3)	0.599 (3)	0.045 (5)*	
H3N	0.1270 (18)	0.837 (3)	0.503 (3)	0.052 (6)*	

### Atomic displacement parameters (Å^2^)

**Table:**

	*U*^11^	*U*^22^	*U*^33^	*U*^12^	*U*^13^	*U*^23^
C1	0.0360 (7)	0.0310 (8)	0.0390 (7)	0.0001 (6)	0.0165 (6)	0.0030 (6)
C2	0.0376 (8)	0.0324 (7)	0.0484 (8)	−0.0050 (7)	0.0086 (7)	−0.0025 (7)
C3	0.0317 (7)	0.0412 (9)	0.0502 (9)	−0.0028 (6)	0.0038 (6)	0.0022 (7)
C4	0.0359 (8)	0.0308 (7)	0.0362 (7)	0.0028 (6)	0.0146 (6)	0.0036 (5)
C5	0.0417 (9)	0.0318 (8)	0.0459 (9)	−0.0015 (7)	0.0057 (7)	−0.0045 (7)
C6	0.0344 (7)	0.0372 (9)	0.0495 (9)	0.0017 (6)	0.0030 (6)	−0.0006 (6)
N1	0.0581 (10)	0.0410 (8)	0.0550 (9)	0.0039 (8)	0.0179 (8)	0.0072 (8)
N2	0.0387 (8)	0.0359 (7)	0.0399 (7)	0.0054 (6)	0.0152 (6)	0.0053 (6)
N3	0.0447 (9)	0.0421 (8)	0.0486 (9)	−0.0043 (6)	0.0154 (7)	−0.0024 (7)
O1	0.0491 (8)	0.0516 (7)	0.0924 (11)	0.0044 (6)	0.0430 (7)	0.0048 (7)
O2	0.0521 (8)	0.0368 (7)	0.0682 (8)	0.0014 (5)	0.0194 (6)	−0.0139 (6)
O3	0.0643 (9)	0.0538 (9)	0.0795 (11)	0.0092 (7)	0.0214 (7)	−0.0178 (8)
O4	0.0622 (8)	0.0551 (8)	0.0420 (6)	0.0030 (7)	0.0033 (6)	−0.0058 (6)
O5	0.0586 (8)	0.0551 (8)	0.0402 (6)	0.0095 (6)	0.0105 (5)	−0.0017 (6)
S1	0.0357 (2)	0.0304 (2)	0.0475 (2)	0.00160 (14)	0.01862 (14)	−0.00021 (15)

### Geometric parameters (Å, °)

**Table:**

C1—C2	1.380 (2)	N1—S1	1.5911 (19)
C1—C6	1.382 (2)	N1—H1A	0.81 (3)
C1—S1	1.7737 (16)	N1—H1B	0.75 (3)
C2—C3	1.386 (2)	N2—H1N	0.93 (3)
C2—H2	0.9300	N2—H2N	0.92 (2)
C3—C4	1.372 (2)	N2—H3N	0.83 (3)
C3—H3	0.9300	N3—O3	1.232 (2)
C4—C5	1.382 (2)	N3—O4	1.239 (2)
C4—N2	1.457 (2)	N3—O5	1.269 (2)
C5—C6	1.382 (3)	O1—S1	1.4283 (14)
C5—H5	0.9300	O2—S1	1.4277 (14)
C6—H6	0.9300		
			
C2—C1—C6	121.65 (15)	S1—N1—H1A	110.7 (19)
C2—C1—S1	119.47 (12)	S1—N1—H1B	109 (2)
C6—C1—S1	118.87 (13)	H1A—N1—H1B	125 (3)
C1—C2—C3	118.84 (15)	C4—N2—H1N	114.6 (19)
C1—C2—H2	120.6	C4—N2—H2N	111.9 (14)
C3—C2—H2	120.6	H1N—N2—H2N	98 (2)
C4—C3—C2	119.44 (14)	C4—N2—H3N	112.0 (17)
C4—C3—H3	120.3	H1N—N2—H3N	115 (2)
C2—C3—H3	120.3	H2N—N2—H3N	104 (2)
C3—C4—C5	121.88 (15)	O3—N3—O4	121.26 (17)
C3—C4—N2	118.52 (14)	O3—N3—O5	119.70 (17)
C5—C4—N2	119.60 (16)	O4—N3—O5	119.05 (16)
C6—C5—C4	118.85 (16)	O2—S1—O1	119.14 (9)
C6—C5—H5	120.6	O2—S1—N1	107.54 (11)
C4—C5—H5	120.6	O1—S1—N1	106.58 (11)
C5—C6—C1	119.34 (15)	O2—S1—C1	107.43 (8)
C5—C6—H6	120.3	O1—S1—C1	107.05 (8)
C1—C6—H6	120.3	N1—S1—C1	108.78 (8)
			
C6—C1—C2—C3	−1.1 (2)	C2—C1—C6—C5	0.8 (3)
S1—C1—C2—C3	179.76 (14)	S1—C1—C6—C5	179.95 (14)
C1—C2—C3—C4	0.5 (3)	C2—C1—S1—O2	−1.42 (15)
C2—C3—C4—C5	0.4 (3)	C6—C1—S1—O2	179.42 (14)
C2—C3—C4—N2	−179.77 (16)	C2—C1—S1—O1	127.65 (14)
C3—C4—C5—C6	−0.7 (3)	C6—C1—S1—O1	−51.51 (16)
N2—C4—C5—C6	179.48 (16)	C2—C1—S1—N1	−117.54 (15)
C4—C5—C6—C1	0.1 (3)	C6—C1—S1—N1	63.29 (17)

### Hydrogen-bond geometry (Å, °)

**Table:**

*D*—H···*A*	*D*—H	H···*A*	*D*···*A*	*D*—H···*A*
N1—H1A···O2^i^	0.81 (3)	2.33 (3)	2.992 (2)	139 (2)
N1—H1B···O4^ii^	0.75 (3)	2.30 (3)	3.045 (3)	172 (3)
N2—H1N···O5^iii^	0.93 (3)	2.01 (3)	2.866 (2)	151 (3)
N2—H2N···O1^iii^	0.92 (2)	1.97 (2)	2.858 (2)	163 (2)
N2—H3N···O5^iv^	0.83 (3)	1.95 (3)	2.770 (2)	171 (2)

Symmetry codes: (i) *x*, −*y*, *z*+1/2; (ii) *x*, −*y*+1, *z*+1/2; (iii) *x*−1/2, *y*+1/2, *z*; (iv) *x*−1/2, −*y*+3/2, *z*−1/2.
